# Human Antimicrobial Peptides and Proteins

**DOI:** 10.3390/ph7050545

**Published:** 2014-05-13

**Authors:** Guangshun Wang

**Affiliations:** Department of Pathology and Microbiology, College of Medicine, University of Nebraska Medical Center, 986495 Nebraska Medical Center, Omaha, NE 68198-6495, USA; E-Mail: gwang@unmc.edu; Tel.: +1-402-559-4176; Fax: +1-402-559-4077

**Keywords:** antimicrobial chemokines, antimicrobial neuropeptides, antimicrobial proteins, cathelicidin LL-37, defensins, dermcidin, hepcidins, histatins, RNases

## Abstract

As the key components of innate immunity, human host defense antimicrobial peptides and proteins (AMPs) play a critical role in warding off invading microbial pathogens. In addition, AMPs can possess other biological functions such as apoptosis, wound healing, and immune modulation. This article provides an overview on the identification, activity, 3D structure, and mechanism of action of human AMPs selected from the antimicrobial peptide database. Over 100 such peptides have been identified from a variety of tissues and epithelial surfaces, including skin, eyes, ears, mouths, gut, immune, nervous and urinary systems. These peptides vary from 10 to 150 amino acids with a net charge between −3 and +20 and a hydrophobic content below 60%. The sequence diversity enables human AMPs to adopt various 3D structures and to attack pathogens by different mechanisms. While α-defensin HD-6 can self-assemble on the bacterial surface into nanonets to entangle bacteria, both HNP-1 and β-defensin hBD-3 are able to block cell wall biosynthesis by binding to lipid II. Lysozyme is well-characterized to cleave bacterial cell wall polysaccharides but can also kill bacteria by a non-catalytic mechanism. The two hydrophobic domains in the long amphipathic α-helix of human cathelicidin LL-37 lays the basis for binding and disrupting the curved anionic bacterial membrane surfaces by forming pores or via the carpet model. Furthermore, dermcidin may serve as ion channel by forming a long helix-bundle structure. In addition, the C-type lectin RegIIIα can initially recognize bacterial peptidoglycans followed by pore formation in the membrane. Finally, histatin 5 and GAPDH(2-32) can enter microbial cells to exert their effects. It appears that granulysin enters cells and kills intracellular pathogens with the aid of pore-forming perforin. This arsenal of human defense proteins not only keeps us healthy but also inspires the development of a new generation of personalized medicine to combat drug-resistant superbugs, fungi, viruses, parasites, or cancer. Alternatively, multiple factors (e.g., albumin, arginine, butyrate, calcium, cyclic AMP, isoleucine, short-chain fatty acids, UV B light, vitamin D, and zinc) are able to induce the expression of antimicrobial peptides, opening new avenues to the development of anti-infectious drugs.

## 1. Introduction

Host defense antimicrobial peptides (AMPs) are key components of the innate immune system shared by both invertebrates and vertebrates. Invertebrates such as insects and crustaceans do not have adaptive immune systems and innate defense systems serve as the only protective mechanism. It is now appreciated that innate immune systems also play an indispensable role in vertebrates by directly killing invading microbes in the early stage. Later, vertebrate AMPs can also help augment the adaptive immune system to further handle infections. Thus, host defense peptides may have dual role: rapid microbial killing and subsequent immune modulation [1,2,3,4,5,6].

The universality of AMPs is evidenced by the identification of these molecules in a variety of organisms. Over 2,300 such peptides have been isolated and characterized according to the online updated Antimicrobial Peptide Database (APD) [7,8]. As of April 2014, there are 233 AMPs from bacteria (*i.e*., bacteriocins), six from protozoa, 12 from fungi, 306 from plants, and 1,801 host defense peptides from animals. AMPs are usually gene-coded and can be constitutively expressed or induced to fend off invading pathogens. In addition, some bacteria use a second mechanism to assemble peptide antibiotics by using a multiple-enzyme system, leading to distinct chemical modifications not observed in gene-coded cases. Most of the AMPs are cationic and short with less than 50 amino acids. Such features are ideal to target the negatively charged surface of bacteria [1,2,3,4,5,6].

AMPs also protect humans from microbial infection. They have been identified in a variety of exposed tissues or surfaces such as skin, eyes, ears, mouth, airways, lung, intestines, and the urinary tract. While human cathelicidin LL-37 is detected in the skin of new born infants [9], human beta-defensin 2 (hBD-2) is frequently expressed in older individuals. Human S100 proteins, hBD-2, human beta-defensin 3 (hBD-3), and cathelicidin are significantly higher in fetal keratinocytes than in postnatal skin cells [10]. In addition, psoriasin (S100A7), RNase 7, and hBD-3 are differentially expressed in healthy human skin [11]. When the skin barrier is broken, psoriasin is up-regulated [12]. Lysozyme and lactoferrin have been found in human tears [13]. In addition, β-defensins are expressed in human middle ear epithelial cells [14]. Drosomycin-like defensin (DLD) [15] is produced in human oral epithelial cells as part of host defense against fungal infection. Defensins, cathelicidins, and histatins are important in preventing oral cavity [16]. Apart from antimicrobial activity, human AMPs such as cathelicidins and defensins possess other functions such as immune modulation, apoptosis, and wound healing [1,2,3,4,5,6]. It is now recognized that certain defensins also play a critical role in sperm fertilization [17,18,19]. By the time this manuscript was completed, 103 human AMPs were found in the APD [7,8]. A selected set of these peptides is provided in Table 1. The lengths of these human peptides range from 10 (neurokinin A) to 149 amino acids (RegIIIα). Their net charges vary from −3 (β-amyloid peptide) to +20 (antimicrobial chemokine CXCL9). On average, human AMPs have 55 amino acids with a net charge of +5.6. Thus, both the average length and net charge for the current list of human AMPs are higher than those averages from all the AMPs (32.4 residues and net charge +3.2). An important reason for this is the inclusion of human antimicrobial proteins (> 100 amino acids) with high net charges (on average +10). The sequence diversity of human AMPs directly determines their structural and functional diversity. This review article highlights the discovery, activity, structure, mechanism of action, and therapeutic strategies of human host defense peptides selected from the APD.

**Table 1:** Discovery timeline of select human antimicrobial peptides and proteins ^1^.

Year	Name	Sequence	Source	Activity ^2^	Ref.
1922	Lysozyme	KVFERCELARTLKRLGMDGYRGISLANWMCLAKWESGYNTRATNYNAGDRSTDYGIFQINSRYWCNDGKTPGAVNACHLSCSALLQDNIADAVACAKRVVRDPQGIRAWVAWRNRCQNRDVRQYVQGCGV	saliva, tears, intestine	G, F	[20]
1985	α-Defensin HNP-1	ACYCRIPACIAGERRYGTCIYQGRLWAFCC	Neutrophils, bone marrow	G, V, F, P, C	[21]
1985	α-Defensin HNP-2	CYCRIPACIAGERRYGTCIYQGRLWAFCC	Neutrophils, bone marrow	G, V, F, C	[21]
1985	α-Defensin HNP-3	DCYCRIPACIAGERRYGTCIYQGRLWAFCC	Neutrophils, bone marrow	G, V, F, C	[21]
1988	Histatin 1	DSHEKRHHGYRRKFHEKHHSHREFPFYGDYGSNYLYDN	saliva	F	[22]
1988	Histatin 3	DSHAKRHHGYKRKFHEKHHSHRGYRSNYLYDN	saliva	G, F	[22]
1989	α-Defensin HNP-4	VCSCRLVFCRRTELRVGNCLIGGVSFTYCCTRV	neutrophils	G, V, F	[23]
1990	RNase 2	KPPQFTWAQWFETQHINMTSQQCTNAMQVINNYQRRCKNQNTFLLTTFANVVNVCGNPNMTCPSNKTRKNCHHSGSQVPLIHCNLTTPSPQNISNCRYAQTPANMFYIVACDNRDQRRDPPQYPVVPVHLDRII	eosinophils	V, P	[24]
1990	RNase 3 (Eosinophil cationic protein, ECP)	RPPQFTRAQWFAIQHISLNPPRCTIAMRAINNYRWRCKNQNTFLRTTFANVVNVCGNQSIRCPHNRTLNNCHRSRFRVPLLHCDLINPGAQNISNCTYADRPGRRFYVVACDNRDPRDSPRYPVVPVHLDTTI	neutrophils	G, V, P	[24]
1992	α-Defensin HD-5	ATCYCRTGRCATRESLSGVCEISGRLYRLCCR	Paneth cells/intestine, female reproductive system	G, V, F	[25]
1993	α-Defensin HD-6	AFTCHCRRSCYSTEYSYGTCTVMGINHRFCCL	Paneth cells/intestine	V, F	[26]
1995	β-Defensin hBD-1	DHYNCVSSGGQCLYSACPIFTKIQGTCYRGKAKCCK	Kidney, Skin, salivary glands	G, F, C	[27]
1995	Cathelicidin LL-37	LLGDFFRKSKEKIGKEFKRIVQRIKDFLRNLVPRTES	neutrophils; skin	G, V, F, P, C	[28,29,30]
1997	β-Defensin hBD-2	GIGDPVTCLKSGAICHPVFCPRRYKQIGTCGLPGTKCCKKP	skin, lung, epithelia, uterus, salivary glands	G, V, F	[31]
1998	Granulysin	GRDYRTCLTIVQKLKKMVDKPTQRSVSNAATRVCRTGRSRWRDVCRNFMRRYQSRVTQGLVAGETAQQICEDLR	cytolytic T and NK cells	G, F, P, C	[32]
1999	Ubiquicidin	KVHGSLARAGKVRGQTPKVAKQEKKKKKTGRAKRRMQYNRRFVNVVPTFGKKKGPNANS	macrophages	G	[33]
2000	Thrombocidin-1 (TC-1)	AELRCMCIKTTSGIHPKNIQSLEVIGKGTHCNQVEVIATLKDGRKICLDPDAPRIKKIVQKKLAGDES	human blood platelets	G, F	[34]
2000	Hepcidin 25 (LEAP-1)	DTHFPICIFCCGCCHRSKCGMCCKT	plasma, Urine/Liver	G, F	[35]
2000	Neuropeptide α-MSH	SYSMEHFRWGKPV	brain	G+, V, F	[36]
2001	β-Defensin hBD-3	GIINTLQKYYCRVRGGRCAVLSCLPKEEQIGKCSTRGRKCCRRKK	Skin, salivary glands	G, V, F	[37]
2001	β-Defensin hBD-4	FELDRICGYGTARCRKKCRSQEYRIGRCPNTYACCLRKWDESLLNRTKP	testis, lung, kidney, neutrophils	G	[38]
2001	Dermcidin	SSLLEKGLDGAKKAVGGLGKLGKDAVEDLESVGKGAVHDVKDVLDSV	eccrine sweat/skin	G, F	[39]
2002	RNase 7	KPKGMTSSQWFKIQHMQPSPQACNSAMKNINKHTKRCKDLNTFLHEPFSSVAATCQTPKIACKNGDKNCHQSHGAVSLTMCKLTSGKYPNCRYKEKRQNKSYVVACKPPQKKDSQQFHLVPVHLDRVL	urinary tract; respiratory tract; skin	G, F	[40]
2003	RNase 5 (angiogenin)	QDNSRYTHFLTQHYDAKPQGRDDRYCESIMRRRGPTSPCKDINTFIHGNKRSIKAICENKNGNPHRENLRISKSSFQVTTCKLHGGSPWPPCQYRATAGFRNVVVACENGLPVHLDQSIFRRPRP	Liver, skin, intestine	G+, F	[41]
2003	Chemokine CCL20	SNFDCCLGYTDRILHPKFIVGFTRQLANEGCDINAIIFHTKKKLSVCANPKQTWVKYIVRLLSKKVKNM	skin	G, F, P	[42]
2003	Chemokine CXCL9	TPVVRKGRCSCISTNQGTIHLQSLKDLKQFAPSPSCEKIEIIATLKNGVQTCLNPDSADVKELIKKWEKQVSQKKKQKNGKKHQKKKVLKVRKSQRSRQKKTT	blood	G, P	[42]
2005	Psoriasin (S100A7)	MSNTQAERSIIGMIDMFHKYTRRDDKIDKPSLLTMMKENFPNFLSACDKKGTNYLADVFEKKDKNEDKKIDFSEFLSLLGDIATDYHKQSHGAAPCSGGSQ	Skin, salivary glands, breast	G-	[43]
2006	RegIIIα	EEPQRELPSARIRCPKGSKAYGSHCYALFLSPKSWTDADLACQKRPSGNLVSVLSGAEGSFVSSLVKSIGNSYSYVWIGLHDPTQGTEPNGEGWEWSSSDVMNYFAWERNPSTISSPGHCASLSRSTAFLRWKDYNCNVRLPYVCKFTD	intestine	G+	[44]
2008	Substance P	RPKPQQFFGLM	the nervous system	G, F	[45]
2008	Drosomycin-like defensin (DLD)	CLAGRLDKQCTCRRSQPSRRSGHEVGRPSPHCGPSRQCGCHMD	oral epithelial cells, skin	F	[46]
2009	Elafin	AQEPVKGPVSTKPGSCPIILIRCAMLNPPNRCLKDTDCPGIKKCCEGSCGMACFVPQ	γδ T cells	G, F, V	[47]
2010	β-amyloid peptide 1-42	DAEFRHDSGYEVHHQKLVFFAEDVGSNKGAIIGLMVGGVVI	brain	G, F	[48]
2011	Chemerin	ELTEAQRRGLQVALEEFHKHPPVQWAFQETSVESAVDTPFPAGIFVRLEFKLQQTSCRKRDWKKPECKVRPNGRKRKCLACIKLGSEDKVLGRLVHCPIETQVLREAEEHQETQCLRVQRAGEDPHSFYFPGQFAFS	skin	G, F	[49]
2012	Amylin	KCNTATCATQRLANFLVHSSNNFGAILSSTNVGSNTY	pancreatic β-cells	G	[50]
2012	KDAMP	RAIGGGLSSVGGGSSTIKY	eyes	G-	[51]
2013	DEFB114	DRCTKRYGRCKRDCLESEKQIDICSLPRKICCTEKLYEEDDMF	epididymis	G, F	[19]

^1^ Data from the APD [7,8]. For a complete list of human AMPs, please visit the APD website (*http://aps.unmc.edu/AP*) and search in the name field using “human”. ^2^ In the APD, antimicrobial activities against different types of microbes are annotated as below: G, bacteria; G+, Gram-positive bacteria only; G-, Gram-negative bacteria only; F, fungi; V, viruses; P, parasites; C, cancer cells.

## 2. Identification of Human Antimicrobial Peptides

Antimicrobial substances might have been noticed long time ago [1,2,3,4,5,6]. Human lysozyme (130 amino acids), discovered in saliva by Alexander Fleming in 1922 [20], is recognized as the first antimicrobial protein [6]. However, the isolation and characterization of many more AMPs with defined amino acid sequences did not start until the 1980s (please refer to the annual AMP discovery plot in ref. [52]). Two major methods were utilized for AMP identification. Initially, chromatographic approaches were used to isolate and characterize new peptides. With the recognition of peptide sequence motifs, bioinformatic approaches were later developed to identify AMPs at the genomic level. In the following, we describe the discovery of the major families of human antibacterial peptides identified during 1985 and 2013.

### 2.1. Human Defensins

Host defense peptides are usually expressed as precursor proteins and the mature form is released by protease processing. In 1985, the Lehrer group isolated a family of the mature form of α-defensins from human blood [21]. Based on the source, property and size, these peptides were named as human neutrophil peptides (HNP-1, HNP-2, and HNP-3). The three defensins have nearly identical amino acid sequences (Table 1). Compared to HNP-2, both HNP-1 and HNP-3 contain only one additional amino acid residue at the N-terminus: alanine for HNP-1 and aspartate for HNP-3. This additional acidic residue in HNP-3 may make HNP-3 less active than HNP-1 or HNP-2 in killing *Staphylococcus aureus*, *Pseudomonas aeruginosa*, and *Escherichia coli*. A fourth human neutrophil defensin, HNP-4, was reported in 1989 [23]. It was also purified to homogeneity by chromatographic methods. HNP-4 has a distinct peptide sequence with 33 amino acids. *In vitro*, purified HNP-4 was shown to kill *E. coli*, *Streptococcus faecalis*, and *Candida albicans*. Lehrer and colleagues found that HNP-1, HNP-2, and HNP-3 are abundant in bone marrow, and can be detected in peripheral blood leukocytes, spleen and thymus by RT-PCR [53].

In 1989, Ouellette and colleagues identified α-defensin genes from mouse Paneth cells [54]. Bevins hypothsized that such orthologs also exist in human epithelial cells as part of the host defense mechanism. A genetic approach was developed to map additional defensin genes based on the high conservation of the nucleotide sequences in the signal coding region as well as the untranslated 5′ region. Using the probes from the conserved regions, they cloned the gene of HD-5 from human Paneth cells [25]. Likewise, HD-6 was identified [26]. These peptides are tissue-specific as they are only expressed in the Paneth cells of human intestines. The six human α-defensins (Table 1) share the same disulfide bond pattern. If we number the six cysteines in Roman numbers: I, II, III, to VI, the three disulfide bonds in α-defensins are C^I^–C^VI^, C^II^–C^IV^, and C^III^–C^V^. Interestingly, alpha defensins have been found in the neutrophils of rabbits, rats, hamsters, and guinea pigs, but not in mice or pigs [55].

Members from the human β-defensin family were discovered in the 1990s. Different from α-defensins, the three disulfide bonds in β-defensins are C^I^–C^V^, C^II^–C^IV^, and C^III^–C^VI^. Also, β-defensins have a slightly longer sequence to allow for an additional helical region. The first human β defensin (hBD-1) was reported by Bensch *et al*. from human plasma in 1995 [27]. Quantitative mRNA analysis revealed kidney as the major source for hBD-1 [56]. In addition, different truncated forms of hBD-1 were also isolated and found to be active against *E. coli*. The activity of hBD-1 might have been compromised in cystic fibrosis (CF) lung due to its salt-sensitive activity against *P. aeruginosa* [57]. Subsequently, hBD-2 was identified from lesional psoriatic skin using the whole *E. coli* affinity column [32]. This material was selected for AMP isolation based on the fact that patients with lesional psoriatic skin have fewer skin infections than expected. This peptide is effective in killing Gram-negative bacteria *E. coli*, *P. aeruginosa*, and yeast *C. albicans*, but is only bacteriostatic against Gram-positive *S. aureus*. Like hBD-1, the activity of HBD-2 is also salt-sensitive [58]. In 2001, both hBD-3 and hBD-4 were documented [37,38]. Based on this disulfide bond linkage pattern, hBD-3 was also identified by using a bioinformatic approach [59,60]. In contrast to hBD-1 and hBD-2, hBD-3 remained active against *S. aureus* and vancomycin-resistant *Enterococcus faecium* at physiological salt concentrations [37]. Bioinformatic studies led to the identification of 28 additional human and 43 mouse β-defensin genes in the respective genome [61]. Several of these β-defensins (hBD-6, hBD-26, hBD-27, hBD-28, and DEFB114) are indeed antimicrobial *in vitro* [62,63,64]. This sequence motif-based peptide prediction may be applied to any other species with a completed genome.

In insects, the activation of TOLL directly leads to the expression of drosomycin against fungal infection [65]. In 2008, human drosomycin-like defensin was detected in oral mucosa [46,66], indicating that this ancient innate defense mechanism is conserved. Sequence alignment in the APD revealed 40% similarity to insect drosomycin, which comprises one α-helical and three β-strands. Therefore, such a combined structure resembles human β-defensins to some extent. This peptide appears to be specifically effective against filamentous fungi (e.g., *Aspergillus* spp) as it did not kill tested yeast, Gram-positive or Gram-negative bacteria. Although the connection pattern of the six cysteines has not yet elucidated, the resulting three disulfide bonds are critical for the antifungal activity of human drosomycin-like defensing [46].

It is interesting to mention that a different type of defensins (called θ-defensins) has been identified from non-human primates [66,67]. These 18-residue defensins are circular due to the formation of a peptide bond between the N- and C-terminal ends. Like α- and β-defensins, they are also stabilized by three sets of disulfide bonds (C^I^–C^VI^, C^II^–C^V^, and C^III^–C^IV^). These defensins are generated by liganding two truncated α-defensins. These genes are not expressed in humans due to the existence of a premature stop codon. Like monkey θ-defensins, synthetic peptides corresponding to these human counterpart pseudogenes are HIV-1 inhibitory [68,69,70]. It is uncertain whether the loss of θ-defensins made humans generally more susceptible to HIV-1 infection.

### 2.2. Human Histatins: Two Genes Multiple Peptides

Histatins are a family of AMPs rich in histidines. In 1988, histatins 1, 3, and 5 were isolated from human saliva by size-exclusion chromatography followed by HPLC separation [22]. All three histatins exhibit the ability to kill the pathogenic yeast, *C. albicans*. Subsequently, other histatins were also isolated [71]. However, only histatins 1 and 3 are gene encoded [22,72], since others are the cleaved products of these two peptides. The histatin genes are located on chromosome 4, band q13 and exclusively expressed in human salivary secretions [73].

### 2.3. Human Cathelicidins: One Gene Multiple Peptides

The significance of cathelicidins in protecting humans from infection is established by data from animal models [74,75,76]. Cathelicidin peptides were first isolated in 1989 [77]. The precursor proteins of cathelicidins share a highly conserved N-terminal “cathelin” domain, but have drastically different antimicrobial sequences, ranging from Pro- and Arg-rich peptides, helical peptides, to disulfide-linked sequences [78]. The word “cathelicidin” was originally used to refer to the entire precursor protein. However, it is now accepted as the family name for mature AMPs from the C-terminal region. Another term hCAP-18 is abbreviated from human cationic protein of 18 kDa, representing the precursors prior to the release of cathelicidin peptides. However, these terms are interchangeably used in the literature.

Based on the conserved sequences in the cathelicidin precursors, Agerberth and colleagues cloned the only human cathelicidin gene and predicted the antimicrobial peptide as FALL-39 in analogy to PR-39 discovered in cattle [28]. Using the probes designed based on rabbit CAP-18, Larrick *et al*. also cloned the C-terminal antimicrobial peptide [29]. In the same year, Cowland *et al*. isolated a 19 kDa precursor protein hCAP-18 from human neutrophils [30]. Subsequently, the European group isolated the natural form of the mature human cathelicidin peptide from neutrophils [79]. Since the isolated peptide contains 37 amino acids and starts with a pair of leucines, it was named LL-37, which is two residues shorter than the initially predicted peptide FALL-39 [28]. Interestingly, ALL-38, another form of human cathelicidin peptides, was also characterized in 2003 [80]. This alterative form contains one more alanine at the N-terminus than LL-37. ALL-38 was generated in female vagina due to the action of gastricsin on sperm hCAP-18. Antimicrobial assays revealed a similar activity spectrum for LL-37 and ALL-38 against a panel of bacteria, including *E. coli*, *S. aureus*, *P. aeruginosa*, and *Bacillus megaterium*. Thus, ALL-38, as well as other active peptides such as SgI-29 [81], plays a defense role in the human reproductive system. In addition, LL-37 fragments were isolated from human skin by chromatographic approaches [82]. These fragments have varying activities compared to intact LL-37. Hence, a single human cathelicidin gene has been processed into different forms of active peptides [83]. This phenomenon, however, is not unique to humans. There is precedence that multiple AMPs are programmed in a single plant gene [84]. One possibility for this is that different cathelicidin fragments provide a means to expanding the functional space of the single human cathelicidin gene. A different model is utilized by sheep, horses, and cattle, which produce multiple cathelicidins with varying functions [85,86,87,88].

### 2.4. Human Dermcidin

Besides human cathelicidin LL-37 [89], dermcidin, an anionic defense peptide, was found in human sweat [39]. Unlike human defensins and cathelicidins that are induced under inflammatory and injured conditions, dermcidin is constitutively expressed in human sweat [90]. Furthermore, dermcidin variants as well as fragments were also detected. It appears that the level of dermcidin did not vary between healthy people and infected patients [91]. In addition, dermcidin may be related to other human diseases such as cancer and atherosclerosis [92,93].

### 2.5. Human Hepcidins

Human liver expressed antimicrobial peptide-1 (LEAP-1) was discovered from human blood ultrafiltrate in 2000 [35]. The same peptide was also found by Ganz *et al*. from human urine and named as hepcidin 25 [94]. This liver-synthesized peptide is especially rich in cysteines (32%), leading to four disulfide bonds in a 25-residue peptide. In 2009, the connection pattern of the four disulfide bonds was revised to C7–C22, C10–C13, C11–C19, and C14–C22 [95]. This antimicrobial peptide also plays an important role in ferrous use [96] and single-residue mutations in this molecule are associated with severe juvenile hemochromatosis, a genetic disease of severe iron overload [97]. Unlike LEAP-1, antimicrobial peptide LEAP-2, however, is not involved in the regulation of iron use [98].

### 2.6. Human AMPs Derived from Known Proteins

Some AMPs are derived from known proteins. Park *et al*. isolated buforin I from amphibians in 1996 [99]. Sequence comparison revealed that buforin I is a cleaved fragment of histone H2A. Of interest, this mRNA was also detected in humans, suggesting a possible role of this peptide in antimicrobial defense [100].

The human airways are essential for the exchange of molecules with the environment. It is necessary to guard this channel to prevent the infection of microbes in the air. In 2001, Ganz and colleagues isolated calcitermin primarily targeting Gram-negative bacteria. This 15-residue peptide is derived from the C-terminus of calgranulin C (a S100 protein). It contains three histidines (His9, His11, and His13) at the N-terminus and has the potential to adopt a helical conformation in membranes. These histidines may explain its enhanced activity in acidic buffers (pH 5.4) and in the presence of micromolar concentrations of ZnCl_2_ [101].

Another example for protein-derived AMPs is KDAMP, keratin-derived AMPs, which were identified from bactericidal lysate fractions of human corneal epithelial cells. These molecules are rich in glycines [51]. The glycines appear to be important for killing *P. aeruginosa* as substitution of a string of glycines with alanines reduced peptide potency. A search of the APD reveals that glycine-rich (>25%) AMPs have also been identified in bacteria, plants, insects, spiders, nematodes, crustaceans, fish, and amphibians. Thus, glycine-rich peptides constitute a common molecular design for host defense [102,103,104,105,106,107,108,109,110].

### 2.7. Antimicrobial Chemokines and AMPs from Human Immune Cells

The fact that some AMPs possess chemotactic effects inspired the evaluation of antimicrobial activity of chemokines, which are known for chemotaxis. In 2000, Krijgsveld *et al*. found antimicrobial thrombocidins, peptides derived from CXC chemokines in human blood platelets [34]. In 2003, Oppenheim and colleagues identified 20 antimicrobial chemokines [42] and additional two members (CCL27 and CCL28) were also reported by Hieshima and colleagues [111]. In 2011, antimicrobial activity of chemotactic chemerin was also reported [49]. Further studies are required to establish the *in vivo* relevance of the *in vitro* activity of these chemokines. The common nature of chemokines and AMPs, however, bridges the innate and adaptive immune systems.

Antimicrobial peptides have also been found in other immune cells. In 1999, Hiemstra *et al*. identified ubiquicidin from ribosomal protein S30 in various tissues. This peptide is active against *Listeria monocytogenes*, *S. aureus*, *Salmonella typhimurium*, *and E. coli*. Considering the fact that *L. monocytogenes* can live within cells, the expression of ubiquicidin in macrophages would limit the replication of this bacterium. Interestingly, ribosomal protein S30 is identical in rats, mice and humans [33]. In 1998, granulysin (74 amino acids) was detected in human cytotoxic T cells and natural killer (NK) cells [32]. Similar proteins called NK-lysins are found in other animals [112]. Granulysin is active against Gram-positive and Gram-negative bacteria, and fungi, including mycobacteria. Thus, the human adaptive immune system has incorporated an innate defense molecule for direct disruption of tumor cells or invading microbes. In addition, human γδ T cells produce antimicrobial peptide elafin [47]. Like human secretory leucoprotease inhibitor (SLPI), elafin was initially identified as a protease inhibitor. Elafin shows an inhibitory effect on bacteria, fungi, and viruses [113].

### 2.8. Antimicrobial Neuropeptides

Antibacterial peptides were also isolated from the neuroendocrine system from cattle. Secretolytin corresponds to the C-terminal fragment (residues 614–626) of bovine chromogranin B [114]. This peptide displayed activity against *M. luteus* and reduced the growth of *B. megaterium*. Vasostatin-1 is a 76-residue N-terminal fragment of bovine chromogranin A. This peptide is active against both Gram-positive bacteria and fungi [115]. It is conserved in humans, pigs, horses, mice, rats, and frogs. Catestatin is a 21-residue AMP derived from human chromogranin A [116]. Subsequently, cattle enkelytin, the C-terminal fragment corresponding to residues 209–237 of proenkephalin-A was also found to be antibacterial [117]. A similar proenkephalin system exists in humans although the processing machinery can differ [118]. Thus, these neuropeptides also play a communication role between neuroendocrine and the immune system [119].

In 1998, human neuropeptide Y (36 residues) was demonstrated to have antimicrobial activity [120]. Interestingly, the antifungal activity of this peptide against *C. albicans* increased several folds by truncating N-terminal 12 residues, implying the importance of the helical region 14–32. A more recent study, however, only observed activity against *E. coli,* but not *C. albicans* [121]. Future studies will clarify whether the peptide has direct antimicrobial effects. In 1999, adrenomedullin, usually expressed on surface epithelial cells, was reported to have antibacterial activity against all the tested bacteria, but not *C. albicans* [122]. Alpha-MSH was added to the human AMP list in 2000 as well [36]. These findings extended host defense peptides to the nervous system [123]. Since then, more antimicrobial neuropeptides have been documented [45,124]. Some of these neuropeptides may become leads for developing new antibiotics. For example, alarin is a human brain neuropeptide with activity against only Gram-negative bacteria such as *E. coli,* but not Gram-positive bacteria such as *S. aureus* [124]. In particular, α-MSH showed *in vitro* antifungal activity against *C. albicans* at fM to pM [125], much lower than nM for some bacterial lantibiotics and µM for many cationic peptides.

### 2.9. Beta-Amyloid Peptides

Beta-amyloid peptides have long been thought to be the culprit of Alzheimer's disease. It is believed that the β-sheet form, not the helical form of the peptide, is toxic. A recent demonstration of antimicrobial activity for β-amyloid peptides adds a new research dimension to this worrisome disease [48]. Further studies along this line could be useful to provide novel insight into the mechanism of this human disease. In 2012, amylin (human islet amyloid polypeptide, hIAPP) was found bactericidal, too [50]. Transformation of amylin into toxic fibrils can disrupt cell membranes and lead to β-cell death, perhaps one of the causative factors of type 2 diabetes mellitus. The aggregated form of amylin is likely to exert its cytotoxicity by damaging cell membranes [126]. It is noticeable that, when in excess, bacteria microcin E492 can form fibrils as a storage form and lose antimicrobial activity [127]. Is there anything in common here from bacteria to humans? Perhaps, this is one mechanism that nature attempts to remove the toxic effects of an over expressed protein, including antimicrobial peptide.

### 2.10. Human Antimicrobial Proteins

There are 14 antimicrobial proteins (>100 amino acids) in the APD database as of March 2014 [8]. This includes the first antimicrobial protein lysozyme [20]. Several eosinophil proteins were purified in 1990. Both eosinophil-derived neurotoxin (EDN, or RNase 2) and eosinophil cationic protein (ECP, or RNase 3) possess anti-parasitic activity against *Brugia pahangi* and *Brugia malayi* [24]. These two ribonucleases are also active against respiratory syncytial virus (RSV) with a single-stranded RNA [128]. In addition, RNase 3 has a unique bacterial agglutinating activity [129]. In 2002, RNase 7 was identified from human skin [40]. Remarkably, it is active against bacteria such as *Mycobacterium vaccae* and yeast, even at 4 °C [130]. Recombinant RNase 7 exhibited antimicrobial activity against uropathogens (*E. coli*, *P. aeruginosa*, *Klebsiella pneumonia*, *Proteus mirabilis*, *E. faecalis*, and *Staphylococcus saprophyticus*) [131,132]. In fact, RNase 7 is the most abundant innate defense peptide in the human urinary tract [132], although the contributions of other human AMPs cannot be ignored [56,133,134,135]. Human RNase 5, (also known as angiogenin or ANG) with weak RNase activity, is initially implicated in angiogenesis. It appears that the nucleus location is necessary for angiogenesis [136]. In 2003, RNase 5 was found to have a toxic effect on Gram-positive bacteria and fungi [41]. However, another study found little activity using a commercial material [137]. In 2006, RNase 8 was found to inhibit *M. vaccae* [138,139]. Therefore, of the eight human ribonucleases, RNases 2, 3, 5, 7, and 8 appear to play a role in host defense.

Psoriasin is another human protein with multiple functions. It is identical to S100A7, a protein member of the S100 family. Psoriasin was initially characterized as a Ca^2+^ binding protein with chemotactic property from human skin psoriatic lesions [140,141]. Subsequently, psoriasin was found in cancer lesions, making it a potential cancer biomarker [142]. However, the antimicrobial activity of psoriasin against *E. coli* was not demonstrated until 2005 [43].

Three RegIII (regenerating gene family protein III) proteins were initially identified in mice [143]. RegIIIα, a RegIIIγ homolog protein, was found in humans. Different from RegIIIβ from mice, human RegIIIα only inhibited the growth of Gram-positive bacteria such as *L. monocytogenes, Listeria innocua*, and *E faecalis* [44].

## 3. Antimicrobial and Anticancer Activities of Human Antimicrobial Peptides

### 3.1. Antibacterial Activities

Antibacterial activities of human AMPs have been mentioned in the preceding section. This section provides an overview of the activity spectrum of these peptides. Based on the APD [8], the majority of human AMPs (90 out of 103) can inhibit the growth of bacteria. They display a broad-spectrum activity against a variety of Gram-positive and Gram-negative bacteria. However, three human AMPs kill primarily Gram-positive bacteria. RNase 5 has an effect on *S. pneumonia* [41], while α-MSH is inhibitory to *S. aureus* [36]. A third AMP, RegIIIα, is active against *L. monocytogenes*, *L. innocua*, and *E. faecalis* [44]. In addition, ten human AMPs are inhibitory mainly to Gram-negative bacteria. These include hBD-26, hBD-27, human calcitermin, psoriasin/S100A7, CCL8, CCL13, CCL19, alarin, HMGN2, and KDAMP peptides [42,43,51,63,101]. They may control different Gram-negative pathogens. For example, both calcitermin and HMGN2 were active against *E. coli* and *P. aeruginosa* [101,144]. Alarin is active against *E. coli*, while the KDAMP peptides are primarily active against *P. aeruginosa*. Thus, these peptides may form the basis for developing new antimicrobials with a desired antibacterial activity spectrum.

### 3.2. Antiviral Activity

Of the 103 human AMPs, 16 are virucidal. They include the six well-characterized human α-defensins (HNP-1, HNP-2, HMP-3, HNP-4, HD-5, and HD-6), three β-defensins (hBD-1, hBD-2, and hBD-3), cathelicidin LL-37, histatin 5, α-MSH, elafin, SLPI, CXCL12, RNase 2, and RNase 3. Elafin is the major antiviral protein in cervicovaginal lavage fluid [113]. Both RNase 2 and RNase 3 inhibit respiratory syncytial viruses (RSV) [145,146]. SLPI levels in saliva and semen, but not breast milk, approximate the level required for HIV-1 inhibition *in vitro* [147]. Although HNP-1 is a lectin that binds to gp120 and CD4, it shows an inhibitory effect after HIV-1 entry [148]. It also inhibits non-enveloped BK virus infection by aggregating virions and blocks binding to host cells [149]. Of the four human β-defensins, hBD-2 and hBD-3 can be induced by viral infection and block HIV replication by directly neutralizing the virions and through modulation of the CXCR4 coreceptor [150]. Human cathelicidin LL-37 is also inhibitory to HIV-1 [151,152]. Wang *et al*. further dissected the active region of LL-37. While the central fragment GI-20 showed an optimal therapeutic index, the LL-37 core peptide FK-13 contains the minimal anti-HIV sequence [152]. In the histatin family, only a histatin-5-derived peptide has an effect on HIV-1 [153]. For a systematic review of AMPs with known anti-HIV activity, interested readers may refer to a recent review article [154].

### 3.3. Antifungal Activity

In the APD, 58 human AMPs are fungicidal [8]. Typical examples are human α-defensins, cathelicidin LL-37, hepcidins, and histatins. In the case of LL-37, protease processing into fragments such as KS-30 and RK-31 is essential in inhibiting *C. albicans* [155]. In addition, the antifungal activity of LL-37 depends on both media and pH. Both LL-25 and RK-31 can rapidily enter the cell cytoplasm [156]. It is likely that these LL-37 fragments target intracellular molecules. It appears that such cathelicidin fragments in human sweat play a role in human skin innate defense against fungal infection [155].

### 3.4. Antiparasitic Activity

Some human AMPs also have antiparasitic properties. These include HNP-1, LL-37, granulysin, CCL2, CCl20, CCL28, CXCL4 (hPF4), CXCL6, CXCL9, CXCL10, RNase 2, and RNase 3. RNases 2 and 3 might be the earliest AMP examples from humans that were demonstrated to have antiparasitic activity [24]. Other more recent examples are HNP-1 against the promastigotes and amastigotes forms of *Leishmania major* [157], chemokine CCL28 against *Leishmania mexicana* [158], LL-37 against *Entamoeba histol* ytica [159], and Platelet factor 4 (hPF4) against malarial parasite *Plasmodium falciparum* [160]. These examples verify that human defense peptides also play a role in controlling parasite infections.

### 3.5. Anticancer Activity

There is a growing interest in developing AMPs into anticancer peptides. Magainins, cecropins, and defensins were all shown to have anticancer effects [161,162,163]. Many other AMPs possess this activity as well and some were discussed in previous review articles [164,165,166]. A more complete and updated list of anticancer AMPs can be searched in the APD database [8]. The 166 anticancer peptides cover multiple sources, including animals (105 peptides), plants (48 AMPs), bacteria (seven bacteriocins), fungi (one peptide), and laboratory synthesis (five peptides).

The anticancer activities of human AMPs have not been widely evaluated since only six members are annotated as anticancer in the APD [8]. They are HNP-1, HNP-2, HNP-3, hBD-1, LL-37, and granulysin. Indeed, reduced expression of granulysin in patients is correlated with the progression of cancer [167]. However, the level of granulysin increased substantially in cancer cells [168]. Similar observations have been made with human defensins and LL-37. While HNP-1 inhibits the growth of human lung adenocarcinoma xenograft in nude mice [169], the same molecule can be overexpressed in tumors [170,171]. HBD-1 can suppress urological and prostate cancers [172,173]. In oral squamous cell carcinoma, hBD-1 also suppressed tumor proliferation, whereas hBD-2 and hBD-3 showed an opposite effect [174]. Likewise, human cathelicidin LL-37 is overexpressed in breast, ovarian, and lung cancers, but it suppresses tumorigenesis in gastric cancer [175]. Such a complex involvement of AMPs in various cancers deserves additional studies. In particular, it is important to elucidate the factors that trigger the overexpression of AMPs in certain cancer cells.

Our current knowledge, however, may be utilized to our advantage. For instance, the over-expression of human cathelicidin LL-37 or defensins may serve as useful biomarkers for cancer diagnosis [176,177,178]. In addition, the fragments of LL-37 with demonstrated anticancer effects *in vitro* [179] and *in vivo* [180] might constitute useful templates for designing new anti-tumor drugs, especially those resistant to existing therapeutics. Anticancer AMPs can work by different mechanisms. The effects may result from a direct bacterial killing when bacteria could be the culprit of cancer (e.g., gastric cancer) [175]. AMPs may selectively kill cancer cells in part due to exposed anionic phosphatidylserines (PS) [181]. It is also possible that some AMPs kill cancer cells indirectly by inducing apoptosis (see below).

### 3.6. Cytotoxic Effects of Human AMPs

It is accepted that many AMPs target bacterial membranes. While bacteria are abundant in anionic lipids such as phosphatidylglycerols (PG) and cardiolipin (CL) [83], human cells comprise zwitterionic phosphocholines (PC) and cholesterol. The differences in membranes of bacterial and human cells to a large extent determine cell selectivity of cationic AMPs. Indeed, among the 103 human AMPs, only three peptides are annotated to have cytotoxic effects on mammalian cells. In the case of LL-37, we found it possible to reduce the peptide cytotoxicity by decreasing peptide hydrophobicity [179,182]. The relatively low cytotoxicity of human AMPs makes them attractive templates for engineering new antimicrobials.

In addition to membrane differences, human cells could use other mechanisms to reduce or remove the potential toxic effect of AMPs on themselves. By expressing a peptide called p33 on the cell surface, the toxic effects of human LL-37 is masked [183]. Instread of directly secreting AMPs, humans also release exosomes (nanovesicles enriched in host defense peptides) into the urinary tract to keep it sterile [184]. It may be speculated that such exosomes, similar to artificial AMP-containing liposomes, could be an effective way to reduce cytotoxicity to human cells.

### 3.7. Other Biological Functions of Human AMPs

In addition to antimicrobial and anticancer activities, many human AMPs possess other functions such as chemotaxis, apoptosis, and wound healing:

*Chemotactic activity*. Currently, 35 human AMPs have chemotactic properties. Examples are defensins and cathelicidin LL-37. Antimicrobial chemokines were originally identified for chemotactic activity. A clear difference between chemokines and AMPs is the concentration needed for action. While the chemotactic effect needs peptides in the nM-pM range, antimicrobial action usually requires µM peptides [185]. Another difference between chemotactic and antimicrobial activities lies in the molecular target. While AMPs usually target membranes of invading bacteria, the chemotactic effects require the association of peptides to host cell receptors. As one example, human LL-37 achieves its chemotactic ability to monocytes, macrophages, neutrophils, and T cells by binding formyl peptide receptor-like 1 (FPRL-1) [186].

*Apoptosis*. Apoptosis is the process of programmed cell death. Different factors can trigger cell apoptosis. AMPs are one of these factors. Interestingly, apoptosis may be induced or suppressed by the same peptide depending on the biological context. Human LL-37 induces apoptosis in vascular smooth muscle cells, primary airway epithelial cells, oral squamous cell carcinoma SAS-H1 cells, intact rat aorta rings and cultured rat aorta smooth muscle cells, Jurkat T-cells and A549 cells [187,188,189,190,191], while it suppresses this process in keratinocytes and neutrophils [192,193]. As a consequence, elucidation of the mechanism to control apoptosis may offer new therapeutic strategies. A recent interesting finding is that inhibition of human LL-37-induced apoptosis by administrating urothelial glycosaminoglycan (GAG) analogs can prevent the development of interstitial cystitis (IC) in a mouse model [194]. LL-37 could suppress the lipopolysaccharides (LPS)-induced apoptosis of endothelial cells, thereby attenuating lethal sepsis/endotoxin shock [195]. In the case of colon cancer, however, activation of apoptosis suppresses tumorigenesis [196]. It seems that apoptosis requires the major antimicrobial region FK-16 (*i.e*., corresponding to residues 17-32) of human cathelicidin LL-37 [179,180].

*Wound healing*. Human host defense peptides can also promote wound healing, a process of injury repairs. Salivary histatin 2 can enhance fibroblast cell migration, whereas human LL-37 at 1 µM can induce cell migration and promote proliferation [197]. It is proposed that these peptides play a role in fast wound coverage.

In summary, antimicrobial activity is a common property of human AMPs, although the *in vivo* relevance has not firmly established for each polypeptide. Furthermore, human AMPs can perform other functions depending on the biological context, peptide concentration, proteases, and the metabolic state. Under diseased conditions, AMPs may have an opposite effect (e.g., cancer suppression *vs*. progression). Understanding the elegant balance of AMPs in these processes in the healthy state as well as the factors that could tilt the balance to a diseased state may yield useful means for cancer treatment.

## 4. Three-Dimensional Structures of Human Antimicrobial Peptides

Three-dimensional structure of human host defense AMPs are helpful to understand the function of AMPs described above. Many short and linear antimicrobial peptides do not have a folded structure free in solution. However, they may become structured upon interactions with host cells by binding to a specific receptor to trigger the biological responses to microbial invasion. In addition, such AMPs may also adopt a defined structure upon association with bacterial targets such as membranes. The bound structure in either category is not trivial to determine. Membrane-mimetic models are normally utilized to determine the membrane-bound structures of receptors or AMPs. These models include organic solvents, detergent/lipid micelles, lipid bicelles, nanodiscs, and lipid bilayers [198,199]. The known 3D structures of these short peptides are primarily determined by multi-dimensional solution nuclear magnetic resonance (NMR) spectroscopy [199]. Some AMPs possess a folded structure in aqueous solutions primarily due to the structural stabilization of disulfide bonds. The structures of these small proteins can be determined by NMR or X-ray crystallographic methods. When both methods are applied, similar structures are usually found. In addition, NMR measurements can gain insight into protein dynamics (*i.e*., motions). Among the 103 human AMPs annotated in the APD database, 42 have a known 3D structure (27 determined by NMR and 15 by X-ray) [8].

Although there are different classification schemes [1,2,3,4,5,6,200], the structures of natural AMPs fall into four large families (α, β, αβ, and non-αβ) [201]. Peptides in the α family contain α-helical structure as the major secondary structure. Typical examples are human cathelicidin LL-37, histatins, dermcidin, and granulysin. The β family is characterized by at least a pair of two β-strands in the structure. Human α-defensins, hepcidins, and SLPI use this type of structure. The αβ family contains both α and β secondary structures, whereas the non-αβ family has neither α nor β structure (also called extended structures). While there are multiple structural examples for the αβ family (Table 2), no structural example has been found for the non-αβ family of human AMPs. In the following, we highlight atomic structures of human AMPs from the α, β, and αβ families. These structures are annotated in the APD database [8] and structural coordinates can be obtained from the Protein Data Bank (PDB) [202] via the APD links.

**Table 2:** Properties of selected human antimicrobial peptides with known 3D structure ^1^.

APD ID	Peptide name	Length	Net charge	Pho%	Boman index	Structure class
2257	Lysozyme	130	+8	40	2.28	α
505	Histatin 5	24	+5	8	4.81	α
780	Lactoferricin	49	+10	36	3.14	α
310	LL-37	37	+6	35	2.99	α
433	Dermcidin	47	−2	38	1.11	α
1161	Granulysin	74	+11	33	3.5	α
2072	Psoriasin/S100A7	101	−1	32	2.3	α
1676	β-Amyloid peptide 1-42	42	−3	45	0.77	α
176	HNP-1	30	+3	53	1.07	β
177	HNP-2	29	+3	51	1.17	β
178	HNP-3	30	+2	50	1.42	β
179	HNP-4	33	+4	51	1.4	β
180	HD-5	32	+4	40	2.6	β
181	HD-6	32	+2	40	1.71	β
192	Hepcidin 20	20	+3	60	0.46	β
193	Hepcidin 25 (LEAP-1)	25	+2	52	0.89	β
2095	SLPI	107	+12	34	1.87	β
451	hBD-1	36	+4	36	1.3	αβ
524	hBD-2	41	+7	36	0.9	αβ
283	hBD-3	45	+11	33	2.87	αβ
811	LEAP-2	40	+4	40	2.94	αβ
2067	RNase 5	125	+11	28	2.99	αβ
2073	RNase 7	128	+16	32	2.16	αβ
2071	RegIIIα	149	+1	33	1.77	αβ
2085	CCL1	73	+10	41	2.25	αβ
2086	CCL8	75	+6	37	2.27	αβ
2088	CCL13	75	+11	36	1.89	αβ
2075	CCL20	69	+8	43	1.34	αβ
2187	CCL27	56	+1	41	1.57	αβ
2076	CXCL1	73	+6	38	1.51	αβ
2080	CXCL10	77	+11	36	2.25	αβ

^1^ Obtained from the Antimicrobial Peptide Database (*http://aps.unmc.edu/AP*) [8]. Peptide hydrophobic amino acid content (percent) is represented by pho% in the table. Protein-binding potential [1] was re-named as Boman index in the APD database in 2003.

### 4.1. The α-Helical Family: Histatins, Cathelicidins, Dermcidin, and Granulysin

*Histatins*. Histatins 1, 3, and 5 are active against *C. albicans* and their candidacidal activities are in the following order: histatin 5 > histatin 3 > histatin 1 [203]. Rai *et al*. investigated the relationship between sequence length and activity using histatin 5 as the template. They found that the C-terminal sequence is important [204]. P-113 with 12 residues corresponding to residues 4–15 of histatin 5 retained anti-candida activity [205]. Using histatin 3 as a model, Zuo *et al*. found increased activity when the active sequence was expressed in tandem (repeated once) [206]. However, duplication of the functional domain of histatin 5 did not enhance candidacidal activity [207]. These results suggest that active domain duplication is not necessary a universal strategy for activity enhancement. Histatin 5 can adopt a helical conformation in the presence of membrane-mimetic agents [204]. However, this helical structure did not appear to be essential for candidacidal activity as peptides with a less helical structure (achieved by proline insertion) can be equally active [208]. Histatin 5 contains a consensus sequence, HEXXH, which is known to bind Zinc. Zinc binding can lead to vesicle fusion and helical conformation as well [209]. Zinc binding actually potentiates peptide activity against gram-positive bacteria *E. faecalis* [210]. An analog of histatin 5 was found to bind DNA and have nuclease activity due to the synergistic oxidative and hydrolytic activities of the metal-peptide complex [211].

**Figure 1:**
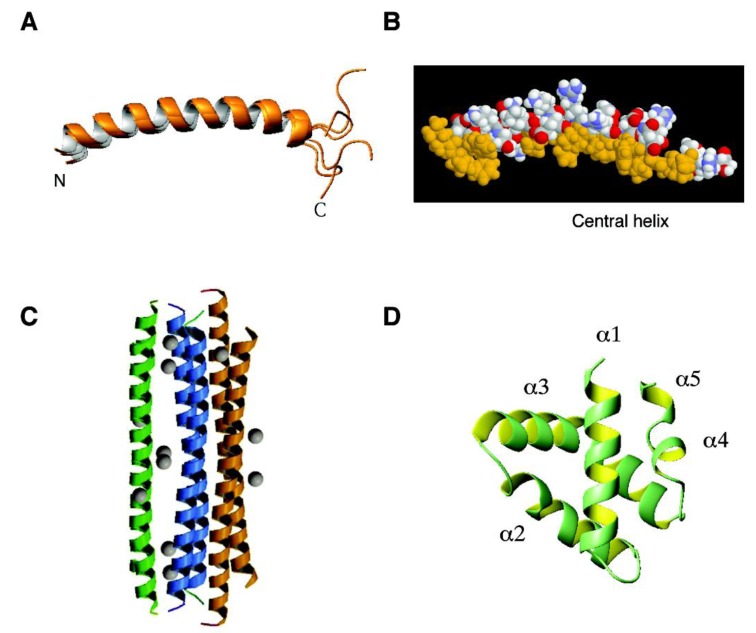
Three-dimensional structures of human antimicrobial peptides from the α-helical family: (**A**) and (**B**) human cathelicidin LL-37 determined by NMR spectroscopy (PDB ID: 2K6O); (**C**) dermcidin determined by X-ray crystallography (PDB ID, 2YMK); and (**D**) granulysin determined by X-ray diffraction (PDB ID: 1L9L). In the case of LL-37, an ensemble of five structures is shown to better view the disordered C-terminal tail (A), whereas a space-filling model is given to show the segregation of the hydrophobic surface (gold) into two domains (B) [182]. The longer one corresponds to the central helix which is important for antimicrobial, anti-biofilm and antiviral activities [83]. Images were generated by using the software MOLMOL [218]. Further details can be found in the text.

*Human cathelicidin LL-37*. To understand the structural basis of antimicrobial activity, 3D triple-resonance NMR spectroscopy was used to solve a high-quality structure for human LL-37 in the presence of sodium dodecyl sulfate (SDS) micelles [182]. LL-37 has a long helix covering residues 2–31, whereas the C-terminal tail is disordered and does not superimpose well to each other (Figure 1A). This structure is fully consistent with the backbone dynamics measured by an independent NMR experiment. This amphipathic helical region determined in SDS micelles is responsible for binding to bacterial outer and inner membranes. Interestingly, the LL-37 tail appeared to be involved in peptide aggregation [212], which influences LL-37 activity [213]. The N-terminal region of LL-37 is less important for antibacterial activity since LL-23, a natural fragment of LL-37, is only active against susceptible *E. coli* or *S. aureus* strains. The weak activity of LL-23 is attributed to a hydrophilic residue Ser9 that splits the hydrophobic face into two clusters [214]. This Ser9 residue also segregates the hydrophobic surface of LL-37 into two hydrophobic domains. It is established that the central helix (Figure 1B) of human LL-37 is critical for antibacterial, anti-biofilm, and antiviral activity (reviewed in ref [83]).

*Dermcidin*. Unlike cationic cathelicidin LL-37 (net charge +6), dermcidin (DCD-1) has a net charge of −2. This peptide also prefers anionic membranes, however. DCD-1L, a variant with one additional leucine at the C-terminus, showed an enhanced affinity for membranes than DCD-1. In the membrane bound state, DCD-1L has a helical conformation. It is located on the membrane surface and can aggregate in the presence of zinc [215]. This oligomeric structure of dermcidin has recently been determined by X-ray crystallography [216]. A hexameric helix-bundle structure (Figure 1C) is proposed to insert into bacterial membrane as an ion channel. In the crystal, the Zn^2+^ coordinates with a group of acidic amino acids. It should be pointed out that the 3D structure of DCD-1L was also determined previously by 3D NMR spectroscopy using a ^15^N-labeled peptide in a 50% trifluoenthanol (TFE) solution. Four helical regions were identified (α1: 5–7; α2: 10–12; α3: 26–33; and α4: 36–45) [217]. In this case, TFE might have disrupted the oligomeric structure of dermcidin. There are precedents for such an effect of TFE. For example, the oligomeric structure of a K^+^ channel did not survive in TFE but retained in membrane-mimetic micelles such as SDS (reviewed in ref. [198]). These examples emphasize the importance of determining the 3D structure of AMPs in a proper environment.

*Granulysin*. Incorporation of AMPs into cytotoxic T cells might have conferred the ability to lyse cells. The crystal structure of granulysin is shown in Figure 1D. There are five helical regions (α1: 2–17; α2: 23–35); α3: 39–61; α4: 66–69; α5: 71–73) [219]. The sequences of human granulysin (74 amino acids) and other NK-lysins share a low degree of similarity (~35%), but homologous modeling reveals antimicrobial features (active helices and basic residue positions) are conserved [112]. Although granulysin only contains two disulfide bonds, it belongs to the saposin-like protein family [220]. Saposin-like proteins comprise several helices usually stabilized by three disulfide bonds [221]. Twelve AMPs in the APD [8] from amoebozoa, nematodes, and large animals such as pigs are annotated to share the saposin-like protein fold [112]. To be antimicrobial, however, neither the entire sequence nor the protein fold is needed. For example, synthetic peptides and analogs derived from helices 3 and 4 are active against *Vibrio cholera* [222]. In addition, the peptide based on the helix-bend-helix motif (residues 31–50) displayed similar antimicrobial activity against Propionibacterium acnes (a key therapeutic target in acne) when synthesized entirely using D-amino acids [223]. Because short peptides can be readily synthesized, they provide useful alternatives for topical treatment of such bacterial infections.

### 4.2. The β Family: α-Defensins

**Figure 2:**
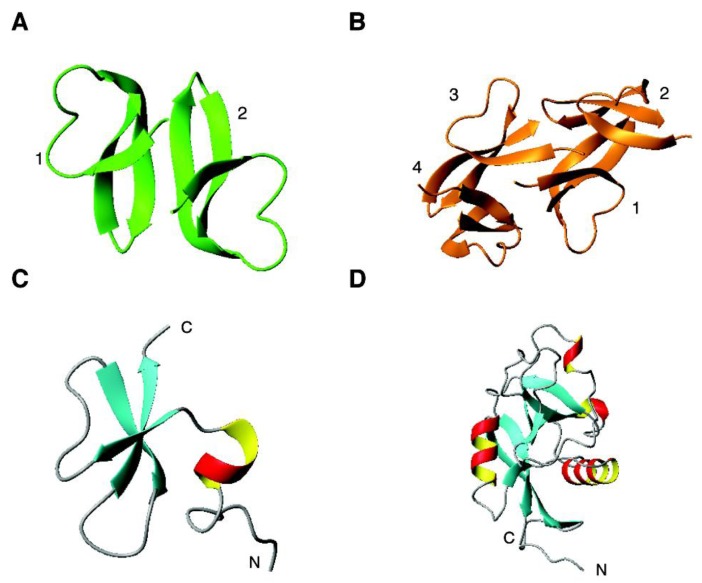
Select 3D structures of human antimicrobial peptides from the β and αβ families: (**A**) HNP-1 (dimeric crystal structure, PDB ID: 3GNY); (**B**) HD-6 (tetrameric crystal structure, PDB ID: 1ZMQ); (**C**) hBD-3 (NMR structure, PDB ID: 1KJ6) and RegIIIα (crystal structure, PDB ID: 4MTH). See the text for further details.

*Alpha-defensins*. Structurally, α-defensins consist of three β-strands that form a β-sheet. In the crystal, a dimeric structure is found for human HNP-1, where two copies of the molecule pack together. HNP-2 and HNP-3 have a similar structure. Thus, it is primarily due to the single amino acid difference in these defensins (Table 1) that influences peptide activity. With a more hydrophobic sequence, HNP-4 is more potent against *E. coli* and *C. albicans* than other human α-defensins [23]. Using a kinetic 96-well turbidimetric procedure, the relative potencies of six human α-defensins were compared. In the case of Gram-positive *S. aureus*, the activity is in the following order: HNP-2 > HNP-1 > HNP-3 > HNP-4. In contrast, their relative potencies against Gram-negative *E. coli* is HNP-4 > HNP-2 > HNP-1 = HNP-3 [224]. Thus, the antibacterial activities of these defensins are also bacteria dependent. This likely reflects the distinct differences in membranes of these organisms. The poor antibacterial activity of HNP-3 is not surprising considering the presence of an acidic aspartate at the N-terminus of the peptide, making it unfavorable to target the negatively charged surface of bacteria. HD-5 displayed a rather potent activity, which is comparable to HNP-2 against *S. aureus* and HNP-4 against *E. coli*. The higher activities of HNP-4 and HD-5 against *E. coli* are correlated with their higher net charge of +4 (Table 2). HD-6 has a poor antibacterial activity. In the crystal, it forms a tetrameic structure (Figure 2B) [123].

### 4.3. The αβ Family: β-Defensins, Antimicrobial Chemokines, RNases, and RegIIIα

*Beta-defensins*. Human β-defensins comprise both α and β structures in the same 3D fold. Figure 2C shows the NMR structure of human β-defensin 3 (hBD-3), which starts with a helical structure followed by three beta strands [225]. NMR translational diffusion studies revealed a dimer for hBD-3, but a monomer for both hBD-1 and hBD-2 in solution. The stronger antibacterial activity of hBD-3 than either hBD-1 or hBD-2 was attributed to the dimeric structure as well as higher charge density on the protein surface [225]. Interestingly, the disulfide-linked form of hBD-1 is poorly active and became highly potent against bacteria and fungus *C. albicans* under reduced conditions where the disulfide-linked structure was disrupted [226]. It seems that the folded hBD-1 is the stored form, which can be transformed into an active form when needed.

**Figure 3:**
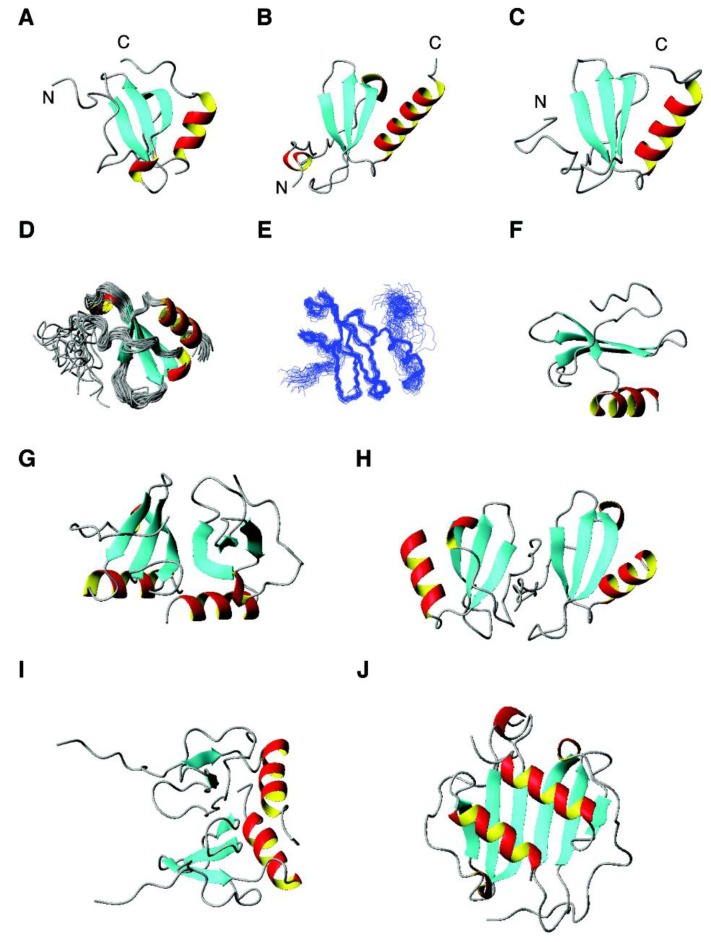
3D structures of human chemokines with antimicrobial activity. Shown are (**A**) CCL1 (NMR structure, PDB ID: 1EL0); (**B**) CCL8 (crystal structure, PDB ID: 1ESR); (**C**) CCL11 (NMR structure, PDB ID: 2EOT); (**D**) CCL21 (NMR structure, PDB ID: 2L4N); (**E**) CCL27 (NMR structure, PDB ID: 2KUM); (**F**) CXCL12 (NMR structure, PDB ID: 2KOL); (G) CCL20 (crystal structure, PDB ID: 1M8A); (**H**) CCL13 (crystal structure, PDB ID: 2RA4); (**I**) CXCL1 (NMR structure, PDB ID: 1MSH); (**J**) CXCL10 (crystal structure, PDB ID: 1O80).

*Antimicrobial chemokines*. Chemokines interact with receptors to realize chemotactic functions. They share a similar fold consisting of a three-stranded sheet followed by one α-helix at the C-terminus [227,228,229]. The N-terminal region is frequently disordered (Figure 3A–C). 

This can be best seen using a superimposed structural ensemble for CCL20 or CCL27 [230,231] determined by NMR (Figure 3D,E). The β-sheet appears to separate the N-terminal domain that interacts with cell receptors and the C-terminal domain that contains the antimicrobial helix for targeting bacterial membranes (Figure 3F) [232]. Some chemokines can also form oligomers. The dimeric forms of CCL20, CCL13, CXCL1, and CXCL10 [233,234,235,236] are shown in panels G to J of Figure 3. The dimer interface is normally composed of the C-terminal helix and strand 3. In the case of CCL13, however, it is the N-terminal region that occupies the interface (Figure 3H). Yung *et al*. found a direct binding of antimicrobial chemokines CXCL9 (net charge of +20) and CXCL10 (net charge +11) to the cell wall of *S. aureus* likely via the positively charged patches on these protein surfaces [237].

Under certain situations, the antimicrobial peptide is generated by further processing of a precursor protein. For example, antimicrobial thrombocidin-1 (TC-1) is produced by truncating two residues from the C-terminus of the parent protein NAP-2 (*i.e*., neutrophil-activating peptide-2), which is poorly active. NMR analysis revealed that the C-terminus of TC-1 is mobile. In contrast, the C-terminus of NAP-2 is less mobile. It was proposed that the additional two residues locked the C-terminus via electrostatic interactions [238]. The additional Asp residue could have masked the positively-charged surface of the C-terminal helix that targets bacterial membranes. Likewise, insertion of an acidic Glu to the N-terminal region of GF-17, a peptide corresponding to the major antimicrobial region of human cathelicidin LL-37 [179], substantially reduced the peptide activity [239].

**Figure 4:**
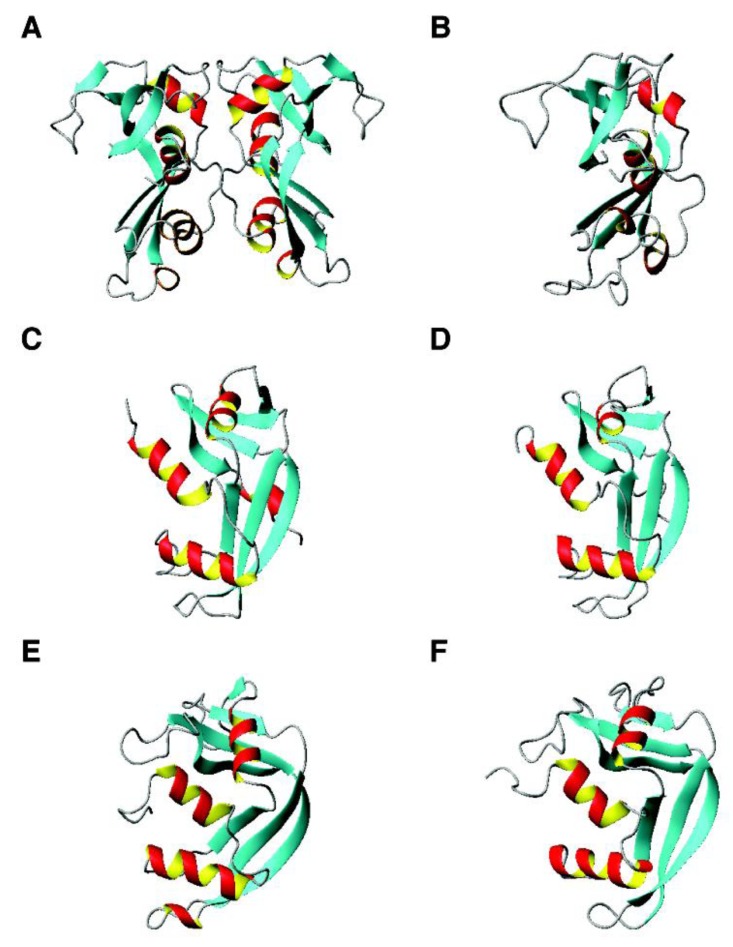
3D structures of human ribonucleases with antimicrobial activity. Shown are (**A**) RNase 3 (dimeric crystal structure, PDB ID: 4A2O); (**B**) RNase 3 (NMR structure, PDB ID: 2KB5); (**C**) RNase 5 (crystal structure, PDB ID: 1B1I); (**D**) RNase 5 (NMR structure, PDB ID: 1AWZ); (**E**) RNase 2 (crystal structure, PDB ID: 2BZZ); and (**F**) RNase 7 (NMR structure, PDB ID: 2HKY).

*RNases*. The structures of RNase 3 (Figure 4, panels A and B) and RNase 5 (panels C and D) were solved by both X-ray diffraction and multi-dimensional NMR spectroscopy. Although RNase 3 is dimeric in the crystal, the protein fold determined by the two techniques is similar. In addition, NMR studies revealed two conformations for His114 of RNase 5 in solution [240]. The structures of RNase 2 and RNase 7 are given in Figure 4 (panels E and F). Unlike chemokines discussed above, the antimicrobial region has been mapped to the N-terminus of RNase 7 [130,241]. In particular, a cluster of lysines were identified as key elements for antibacterial activity (bold in Table 1). It is proposed recently that the N-terminal antimicrobial function is conserved in the ribonuclease family [242]. One may wonder why a protein is created for bacterial defense if only part of the chain is required to kill pathogens. One possibility is the stability gain as part of the protein. Another possibility is that a folded protein structure allows for the incorporation of a variety of active sites on the protein surface. In certain cases, such functional sites may be overlapping [243]. In the case of RNase 7, the adjacent active site and antimicrobial residues allows us to propose a yet-to-be-proved “peel-and-kill” model. In other words, binding to bacteria by the cationic amino acids is followed by digestion of pathogenic nucleic acids. The multiple active sites also enable functional regulation. For example, an endogenous molecule can bind to RNase 7 and regulates its antimicrobial activity [244]. This could be one of the unique features of antimicrobial proteins distinct from small antimicrobial peptides.

*Antimicrobial lectin RegIIIα*. RegIIIα (or HIP/PAP) is a C-type lectin that binds peptidoglycan carbohydrates of Gram-positive bacterial cell walls. The structural basis of this binding has been elucidated (Figure 2D) [245]. Different from other C-type calcium-dependent lectins, the binding of RegIIIα to peptidoglycans is calcium independent (*i.e*., lacking calcium-binding motif). The binding, however, requires the “EPN” motif and depends on sugar chain length. However, it seems that this peptidoglycan binding serves as an early recognition step for the peptide action as it can create a pore on bacterial membranes. The structure of the oligomeric form of the protein has recently been determined by combining X-ray structure and electron microscopy data, providing insight into the lethal step of bacterial killing by this intestine lectin [246].

## 5. Mechanism of Action of Human Antimicrobial Peptides

It is generally believed that cationic AMPs target anionic bacterial membranes. In the past years, significant advances have been made in elucidating the molecular targets of human AMPs. As described below, human AMPs can interact with a variety of molecular targets either on the cell surface (including membranes) or within the cells.

### 5.1. Targeting Bacterial Cell Wall

The molecular targets of AMPs are not limited to bacterial membranes. The assembly of HD-6 on bacterial surface entangles bacteria, providing a new defense mechanism for human innate immunity [123]. HNP-1 targets lipid II to block the biosynthesis of bacterial cell walls [247]. Böhling *et al*. found a close correlation of the hBD-3 activity with cell wall components [248]. A subsequent study corroborated the binding of hBD-3 to lipid II as well [249]. In the APD [7,8], there are 18 AMPs that use this mechanism to combat bacteria. Examples are nisins, mersacidin from bacteria, plectasin from fungi, Cg-Def, an oyster defensin [250,251,252]. Hence, blocking bacterial cell wall synthesis is a widely deployed innate defense mechanism. It is possible to identify small molecule mimetics that bind bacterial cell walls [253,254]. Although not discussed here, other defensins can directly recognize specific lipids in fungal membranes [255,256,257].

RegIII proteins are a family of lectins that can specifically recognize the carbohydrate portion of bacteria. While RegIIIα targets the peptidoglycan carbohydrate backbone for Gram-positive bacterial killing [245], mouse RegIIIβ associates with the lipid A portion of Gram-negative bacterial LPS [258]. In the case of murine RegIIIβ, amino acid residues in two structural motifs termed “loop 1” and “loop 2” are important for peptidoglycan and lipid A binding (Arg-135, Asp-142) and for the bactericidal activity (Glu-134, Asn-136, and Asp-142).

It is well known that human lysozyme not only binds a single peptidoglycan chain but also cuts the sugar repeats, thereby inhibiting bacterial cell wall synthesis [259]. In addition, some AMPs can associate with cell surface proteins to interfere with the docking and entry of viruses such as human immunodeficiency virus type 1 (HIV-1). For example, the association of SLPI with human annexin II can avoid the attachment of viral lipid phosphatidylserine (PS) to the same protein in human macrophages [260].

### 5.2. Targeting Bacterial Inner Membranes

Human cathelicidin LL-37 is a representative member in the helical family. It is proposed that LL-37 disrupts bacterial membranes. The membrane disruption of LL-37 involves at least three steps. First, the cationic peptide can recognize and coat the anionic surface of bacteria. With a classic amphipathic helical structure, this cationic peptide prefers to target anionic bacterial membranes. Second, LL-37 binds to the outer membranes and cross the outer membrane. Third, the peptide reaches the inner membrane. It initially binds to the inner membrane parallel to the surface, which is the basis for the carpet model [261]. At elevated concentrations, the peptide may disrupt the membranes by micellization. Alternatively, the peptide might take a vertical position to form a pore [262].

The ability of hepcidin 25 (hep-25) and its isoform hepcidin 20 (hep-20) to perturb bacterial membranes is markedly pH-dependent. The membrane disruption is more evident at acidic pH than at neutral pH. At acidic conditions, histidines become positively charged and more effective in membrane disruption [263].

While there is no agreement in the case of human LL-37 regarding the carpet or pore formation, recent structural determination of dermcidin provides evidence for possible pore formation in bacteria membranes. In the crystal structure [216], the peptide forms a hexamer, where two trimers are connected by zinc (Figure 1C). It is proposed that this structure might be directly inserted into bacterial membranes, serving as an ion channel.

In addition to α-defensins (HD-5 and HD-6), RegIII peptides are expressed to control the microbiota and keep the bacteria away from the epithelial surface of intestine. In particular, specific bacteria (e.g., *Bifidobacterium breve* NCC2950) can effectively induce the expression of RegIIIγ (an ortholog of human RegIIIα) in the intestine of mice via the MyD88-Ticam1 pathway [264]. RegIIIα is a lectin that binds to peptidoglycans of Gram-positive bacteria. However, it adopts a hexametic membrane-permeating pore structure to kill bacteria [246]. Such a pore is reminiscent of other pore structures solved for toxins [265,266,267]. The structure also provides a basis for selective killing of Gram-positive bacteria such as *L. monocytogenes*, *L. innocua*, and *E. faecalis* but not Gram-negative bacteria. This is because LPS, the major component of the outer membranes of Gram-negative bacteria, inhibits the pore-forming activity of RegIIIα [246].

### 5.3. Cell-Penetrating Peptides and Intracellular Targets

There are other AMPs that may work primarily by binding DNA. Buforin is such an example [83]. This is not surprising because this AMP was derived from DNA-binding histone 2A. In addition, SLPI, a small protein that inhibits elastase and cathepsin G, displayed antibacterial activity against *E. coli* by binding nucleic acids [268]. It is also likely that AMPs kill bacteria by more than one mechanism. For example, human LL-37 may first damage bacterial membranes followed by DNA binding, leading to the shutdown of bacterial machinery [83].

Unlike human LL-37, histatin 5 caused only small membrane damaging effects [269]. To interpret the killing effect of this peptide, two models have been proposed. In the first model, treatment of *C. albicans* with histatin 5 induces the efflux of ATP and increases cell permeability to small molecules, leading to ion imbalance. Thus, *C. albicans* cells respond by activating the osmotic stress responding pathways to minimize ion loss. This model is supported by the fact that knocking out the TRK1 gene that encodes a major K^+^ uptake system made histatin 5 ineffective. A second model was also proposed based on the observation that the candida killing ability of histatin 5 is lost using a mitochondrial respiration mutant or after treatment with sodium azide that inhibits cellular metabolism [270]. In addition to the requirement of the mitochondrial respiration machinery [271], cellular internalization of histatins is also facilitated by peptide binding to heat shock protein Ssa2p on the surface of *C. albicans* [272]. This interference with mitochondrial respiration chain may be responsible for the formation of reactive oxygen species (ROS), leading to cell death [273]. Vylkova *et al*. performed a DNA microarray study of the effect of histatin 5 and found that these two models can be unified. This is because the oxidative stress could be produced as a secondary effect of osmotic stress. This proposal is in line with the observation that the killing of histatin 5 is facilitated in the presence of an osmotic agent sorbital but not an oxidant agent H_2_O_2_ [274]. It should be mentioned that human GAPDH(2–32), an antifungal peptide derived from the highly conserved protein GAPDH, can also enter *C. albicans* to induce apoptosis [275].

As a different mechanism to combat bacterial infection, some AMPs are reported to penetrate immune cells and activate them to boost immune response. For example, chromagranin A-derived peptides can penetrate neutrophils, bind to cytoplasmic calmodulin, and induce Ca^2+^ influx, leading to neutrophil activation and immune system augmentation [276].

Granulysin is an effector molecule in the cytotoxic granules of cytotoxic T lymphocytes and natural killer (NK) cells. It can kill intracellular pathogens in infected cells in the presence of perforin and to induce a cytotoxic effect against tumor cells. Although perforin and granulysin can colocalize [277], it is unclear how they work together in bacterial killing. A recent study reveals that perforin can form pores that preferentially allow the entry of cationic molecules [278]. Thus, granulysin might have entered the cell via the perforin pores, thereby providing yet another mechanism for intracellular bacterial killing by forming a molecular pair.

## 6. Concluding Remarks and Potential Therapeutic Strategies

Human antimicrobial peptides and proteins occupy an important niche in the current research on human host defense and innate immunity [1,2,3,4,5,6,279]. Except for antimicrobial protein lysozyme, which was found in 1922, most of short cationic peptides were discovered after 1980 (Table 1). By the time this article was written, over 100 human AMPs have been identified and characterized. They were either isolated from human tissues or predicted from the human genome by bioinformatics. Although genomic prediction constitutes an invaluable method, isolation from natural sources remains important in determining the exact mature form of AMPs. The discovery story of LL-37 nicely illustrates this point (Section 2.3). These peptides have diverse amino acid sequences (Table 1) and physical properties (Table 2), leading to a panel of defense molecules with varying activities (Table 1). While psoriasin and KDAMP primarily inhibit the growth of Gram-negative bacteria, RegIIIα is mainly active against Gram-positive bacteria. In addition, histatins and drosomycin-like defensin are primarily fungicidal. Many human AMPs such as LL-37 and defensins are broad-spectrum peptides against pathogens.

Remarkably, human AMPs are able to hinder bacterial growth by interactions with different targets, ranging from surface molecules (e.g., cell walls), inner membranes, to intracellular molecules (Table 3). Some AMPs can interact with two or more molecules. For example, the binding of RegIIIα to peptidoglycans constitutes only the initial recognition step and subsequent pore formation in bacterial membranes could be the lethal step [246].

**Table 3:** Select human antimicrobial peptides and their proposed targets.

APD ID	AMP	Structure	Molecular target
181	HD-6	β	Aggregate on bacterial surface
283	hBD-3	αβ	Bacterial cell wall (lipid II)
176	HNP-1	β	Bacterial cell wall (lipid II)
2257	Lysozyme	α	Cell wall carbohydrate
2071	RegIIIα	αβ	Membrane pores
310	LL-37	α	Bacterial membranes and/or DNA
433	Dermcidin	α	Membranes ion channel
2017	hGAPDH(2-32)	Unknown	Intracellular targets of fungi
505	Histatin 5	α	Intracellular mitochondria
2352	Chromagranin A-derived peptides	Unknown	Cytoplasmic calmodulin of neutrophils
1161	Granulysin	α	Perforin generates a pore to allow granulysin to enter the cell and kill intracellular bacteria

For interactions with different molecules, human AMPs are capable of adopting a variety of 3D structures (Figure 1, Figure 2, Figure 3 and Figure 4). It is clearly important to determine the structure to high quality so that the molecular basis of these interactions can be uncovered accurately (reviewed in ref. [52]). It is also important to correlate the structure with the active state of the peptide. In the case of dermcidin, which oligomerizes on bacterial surface, the helix-bundle structure determined by X-ray crystallography [216] should be more relevant. Likewise, the disulfide-bonded structure of HBD-1 does not explain peptide activity under reduced conditions [226]. Therefore, human AMPs are diverse in terms of amino acid sequence, 3D structure, activity, and mechanism of action. 

Many human AMPs are currently under close examination for their functional roles as well as potential applications in detection and diagnosis of human diseases. The type and expression level of human AMPs, if accurately mapped, may have clinical relevance. A clear variation in the expression level of AMPs can serve as biomarkers for human diseases, such as eczema severity and cancer [280,281,282,283]. In addition, this remarkable array of molecules may be used for detection, imaging, and diagnosis of bacterial infection. An example of this application is based on the preferential association of Technetium-99m labeled ubiquicidin with bacteria, enabling the physician to differentiate infection from aseptic loosening of hip prostheses in 30 min with high accuracy [284,285,286].

The collection of human AMPs discussed herein also inspires us in developing novel therapeutics [287]. First, new antimicrobials may be developed using human AMPs as templates. The rationale is that AMPs have remained potent for millions of years and are thus less prone to microbial resistance [1,2,3,4,5,6]. In particular, peptides with different structural scaffolds may kill the same bacterium by different mechanisms (Table 3). Furthermore, the same peptide sequence can be tailored into various peptides that selectively target pathogens such as Gram-positive, Gram-negative bacteria, or viruses [83]. This is highly desirable for selective bacterial elimination without destroying the probiotic microbial flora. The success of this line depends on whether a selected template will achieve the desired potency *in vivo* against a target pathogen, low cytotoxicity to humans, stability to proteases, and cost-effective production [287]. One can also consider alternative peptide forms. For instance, a pro-drug can be used to reduce the cytotoxicity of AMPs if a mechanism can be found to release it when needed [288]. While portions of antimicrobial proteins are preferred to design novel antimicrobials [222,223,242,289], a whole protein may also be considered. Unlike short peptides, the folded structure of proteins confers stability to the action of proteases. However, the production of such a long polypeptide chain may require recombinant expression in bacteria or cell-free systems [199].

Second, new strategies are actively sought to bring the invading pathogens under control. It is appreciated that different receptors and signal pathways are activated in response to the invasion of different microbes [65]. Of outstanding interest is that non-pathogen factors can also induce AMP expression (Table 4). One of the earliest examples might be the light therapy invented by Niel Finsen [290]. The establishment of a link between light therapy, vitamin D and human cathelicidin LL-37 expression provides a completely different way for infection treatment. Instead of treating patients with traditional antibiotics, doctors may be able to use light or vitamin D [291,292]. Indeed using narrow-band UV B light, the level of vitamin D was increased in psoriasis patients (psoriasis is a common autoimmune disease on skin) [293]. In addition, other small molecules such as butyrate can induce LL-37 expression [294]. Components from Traditional Chinese Medicine may regulate the AMP expression as well [295]. These factors may induce the expression of a single peptide or multiple AMPs [296]. It is also possible that certain factors can work together to induce AMP expression. While cyclic AMP and butyrate synergistically stimulate the expression of chicken β-defensin 9 [297], 4-phenylbutyrate (PBA) and 1,25-dihydroxyvitamin D3 (or lactose) can induce AMP gene expression synergistically [294,298]. It appears that stimulation of LL-37 expression by histone deacetylase (HDAC) inhibitors is cell dependent. Trichostatin and sodium butyrate increased the peptide expression in human NCI-H292 airway epithelial cells but not in the primary cultures of normal nasal epithelial cells [299]. However, the induction of the human LL-37 expression may not be a general approach for bacterial clearance. During *Salmonella enterica* infection of human monocyte-derived macrophages, LL-37 is neither induced nor required for bacterial clearance [300].

**Table 4:** Some known factors that induce antimicrobial peptide expression

Factor	AMP induced	Cells	Ref
Bacteria/LPS	LL-37, HBD-2	keratinocytes	[296]
TNF-α	LL-37, HBD-2	keratinocytes	[286]
UV Light	LL-37, HBD-2, chemerin	keratinocytes	[286,301]
Vitamin D3	LL-37	neutrophil progenitors and EBV-transformed B cells	[302,303]
Lactose	LL-37	colonic epithelial cells T84, THP-1 monocytes and macrophages	[304]
Short-chain fatty acids	LL-37;pBD-2, pBD-3, pEP2C, and protegrins	human HT-29 colonic epithelial cells and U-937 monocytic cells;	[305,306]
Isoleucine	hBD-1;epithelial defensins	human colon cells, HCT-116; bovine kidney epithelial cells	[307,308,309]
Arginine	hBD-1	human colon cells, HCT-116	[307]
Ca^2+^	hBD-2, hBD-3	human keratinocyte monolayers	[310]
Zn^2+^	LL-37;pBD-1, pBD-2, pBD-3	Caco-2 cell; Intestinal epithelial cells	[311]
Butyrate	LL-37	colon, gastric and hepatocellular cells	[312]
Albumin	hBD-1	human colon cells, HCT-116	[307]
Cyclic AMP/Butyrate	Chicken β-defensin 9	macrophages and primary jejunal explants	[297]
Phenylbutyrate/1,25-dihydroxyvitamin D3	cathelicidins	immortalized human bronchial epithelial cell line VA10	[298]

Finally, immune modulation peptides may find therapeutic use because they do not act on microbes directly and thereby are less likely to induce antimicrobial resistance [4,5]. Immune modulation is activated via peptide binding to host cell receptors that initiate various signal transduction pathways. Recently, a natural peptide was found to have immune modulating activity but no antimicrobial activity [313]. Besides engineering peptides with distinct properties, there has been growing interest in elucidating the bacterial mechanisms in generating resistance to AMPs or by subverting host immune systems [314,315]. It can be anticipated that new therapeutic approaches will continue to emerge from our understanding of the host-pathogen interactions. All these strategies will facilitate the development of AMPs into novel antimicrobials to meet the challenge of antibiotics-resistant superbugs, RNA viral infections and difficult-to-treat cancers [287]. 
